# Socioeconomic factors and low birth weight in Mexico

**DOI:** 10.1186/1471-2458-5-20

**Published:** 2005-03-03

**Authors:** Laura P Torres-Arreola, Patricia Constantino-Casas, Sergio Flores-Hernández, Juan Pablo Villa-Barragán, Enrique Rendón-Macías

**Affiliations:** 1 Health Services and Epidemiologic Research Unit. National Medical Centre Century XXI, Mexican. Institute of Social Security, Mexico; 2 Health Economics Research Unit. Mexican Institute of Social Security, Mexico; 3 Health Research Coordination. Centre Century XXI, Mexican Institute of Social Security, Mexico; 4 National Adviser. Pan American Health Organisation, Mexico; 5 Public Health Coordination. Mexican Institute of Social Security, Mexico

## Abstract

**Background:**

Low birth weight (LBW) is a public health problem linked to lack of equity in populations. Despite efforts to decrease the proportion of newborns with LBW, success has been quite limited. In recent years, studies focused on explaining how social factors influence this problem have shown that populations with greater inequities have a greater proportion of newborns with LBW.

**Methods:**

The objective was to describe socioeconomic factors related to LBW adjusted by demographic, reproductive and health services variables in Mexico City. A case-control study was carried out in three hospitals with gynaecological and obstetrics services in Mexico City during the first half of 1996. During the recruiting period all children with LBW (cases), defined as newborns weighing <2500 grams, were matched with children born on the same day to control for time of birth. Upon arrival at the hospital for delivery, women were interviewed to determine if they met our inclusion criteria. Women with a history of chronic conditions and those with twin or multiple pregnancies were excluded. Variables with clinical and statistical significance were included in a multivariate model (logistic regression).

**Results:**

We found that low socioeconomic level was the most important risk factor for LBW and was independent of other factors, including those related to reproduction and nutrition, smoking, morbidity during pregnancy, accessibility to health services and prenatal care (OR 2.68; 95% CI 1.19, 6.03).

**Conclusion:**

We found that socioeconomic factors are relevant to LBW. However further research should be done in different population groups as well as developing precise ways of measuring socioeconomic factors and their role in reproductive health.

## Background

On average, the worldwide incidence of low birthweight (LBW) is 17% per year, making LBW an important infant health problem in many populations [1]. The incidence of LBW varies among countries, ranging from 4% to 6% in Western countries like Sweden, France, United States and Canada (UNICEF 2003) and much higher in developing countries. In Latin America, the overall LBW rate varies according to geographical region. The Pan American Health Organization has estimated that the overall LBW rate is 8.27% in South America and Mexico and ranges from 6% in Peru to 10% in Bolivia and Venezuela. In Central America the overall LBW rate is 11.2%, ranging from 6% in Belize and El Salvador to 15% in Nicaragua [2]. The LBW rate can also vary within countries. For example, in Mexico, the incidence ranges from 8.2% to 12%, depending on geographic al region [3].

In addition to its impact on infant mortality, LBW has been associated with higher probabilities of infection, malnutrition and handicapping conditions during childhood, including cerebral palsy, mental deficiencies and problems related to behavior and learning during childhood [4-6]. Children who survive LBW have a higher incidence of diseases, retardation in cognitive development, and undernourishment. There is also evidence that LBW or its determinant factors are associated with a predisposition to higher rates of diabetes, cardiac diseases and other future chronic health problems [7-9].

The biological processes that affect the fetus *in utero *are related to the mother's physiology, including her nutrition (mother's weight before pregnancy and history of having newborns with LBW), exercise, infections, and consumption of tobacco, alcohol and other drugs [10,11].

During the fetal phase, growth depends on the nutritional condition of the mother, indicating that pregnant women should not only increase their weight but also consume essential nutrients. For many women in the developing world, however, economic, social and cultural factors make it difficult for them to obtain the necessary food and healthcare, which are closely interrelated [12].

Associations between poorer child health and poverty, inequity and social exclusion have been documented worldwide and have been shown to be independent of research methods, local culture, and available health care services [13]. While the relationship between socio-economic conditions and health have been of interest and concern for centuries, recent studies have sought to identify the social factors most relevant to health [14]. Some authors consider that health therefore may be an important determinant of opportunities in life and this process, termed "selection by health", and suggests that health "selects" people in different social strata [15,16].

Among the socio-economic factors are income, education, occupation, household leadership and gender differences related to roles within the family [17,18]. In Mexico, there are also differences in socio-economic conditions resulting from geographic area and political organization. These may affect various health parameters, including mortality, morbidity and reproductive behavior.

Several studies have shown different results on whether socioeconomic factors affect pregnancy outcomes and newborn conditions [19-21]. The inconsistency of these findings may be due to poor clarification of the mechanisms by which socioeconomic status affects LBW. This is especially true in relationship to the mother's nutritional conditions, although low maternal weight before pregnancy and small weight gain during pregnancy have been shown to be associated with higher risks of preterm infants and LBW [22-25]. Other studies have questioned whether maternal occupation or educational level is associated with LBW, or whether the latter is related to a group of socioeconomic factors.

In this study, we have analyzed the socio-economic factors related to birthweight adjusted by others known factors in the urban population of Mexico in three hospitals located in three different geographic areas of Mexico City.

## Methods

A case-control study was carried out in three hospitals with gynaecological and obstetrics services in Mexico City during the first semester of 1996. We recruited 154 LBW newborns, defined as newborns weighing <2500 grams, from these three hospitals, as well as 474 controls chosen from births on the same day to control for time of birth. All newborns included in this study provided a statistical power of 80% when we assumed a = 0.05 (one-sided test), a case/control ratio 1:3, and a 3% difference among both groups in the low socioeconomic level [26].

Upon arrival at the hospital for delivery, women were interviewed to determine if they met our inclusion criteria. Women with a history of previous chronic conditions or those with twin or multiple pregnancies were not included. Following delivery, and after obtaining informed consent, each woman was administered a questionnaire by a trained interviewer to obtain information about socio-economic, reproductive, and nutritional factors. Socioeconomic factors included age, level of education, civil status, occupation, income and owning certain goods; reproductive factors included parity, history of preterm delivery and LBW, which were classified as positive or negative for all previous pregnancies; and nutritional factors included calcium and iron supplementation, pre-gestational weight, prenatal care, morbidity during pregnancy and tobacco exposure. Clinical records were also reviewed to verify information about each newborn.

To create a socioeconomic level index, we used two variables that have been considered as proxies [27]. Ownership of goods was defined as to whether a woman owned her house or flat and if she had a car and whether the woman and her partner were employed. Using these two variables, we constructed a three category socio-economic level index, in which High indicated that the woman and her partner had jobs and goods; Medium indicated that the woman or her partner had jobs or goods; and Low indicated that the woman and/or her partner did not have jobs or goods.

Statistical analysis was performed by describing sociodemographic, reproductive and prenatal care characteristics of the mother and newborn. Univariate analysis was used to evaluate the association between the independent variable (socioeconomic level) and covariates with LBW (outcome variable). Stratified Mantel-Haenszel analysis was performed to evaluate confounding and/or interaction (i.e. parity and age). To obtain the association magnitude of the socioeconomic level adjusted by the covariates, multivariate analysis was performed (logistic regression). Variables with clinical and statistical significance were included in multivariate modeling. We used three models to classify factors relevant to LBW into three groups as known, controversial and unknown risk factors for LBW.

Model 1 included socioeconomic level index and maternal age, maternal education, marital status and accessibility to public services. In model 2 we included all variables of the model 1 besides, prenatal care, tobacco exposure, and morbidity during pregnancy were considered as adjusted variables. Finally, model 3 included all variables previously mentioned (model 1 and model 2 besides reproductive variables.

Analyses were performed using StataCorp. 2002. Stata Statistical Software: Release 7.0 College Station, TX:Stata Corporation.

## Results

The sociodemographic characteristics of the LBW and normal birthweight groups are shown in Table 1. There was a high proportion of married women in both groups, and 90% of women in both groups had access to public services, including electricity, water and a sewage system. The effect of maternal education had the expected direction, although it was not statistically significant. Women in the lower socio-economic level had a higher risk for LBW (OR, 2.19; 95% CI, 1.18–4.07) than those in the medium and high socioeconomic levels.

**Table 1:** Sociodemographic characteristics of mothers of low and normal birthweight infants

Variable	Birthweight		OR	95% CI
	**<2500 grs (*n = 158*)**	**≥ 2500 grs (*n = 474*)**		
Socioeconomic level				
**High**	81	267	1.0	
**Medium**	57	177	1.06	[0.71,1.56]
**Low**	20	30	2.19*	[1.18,4.07]
Maternal age (years)				
**<19**	22	83	0.77	[0.46,1.29]
**20–30**	110	321	1.0	
**>30**	26	70	1.08	[0.65,1.80]
Maternal education (years)				
**>12**	13	50	1.0	
**10–12**	56	143	1.50	[0.76,2.98]
**7–9**	52	165	1.21	[0.61,2.40]
**<7**	37	116	1.22	[0.60,2.50]
Marital Status				
**Married**	138	429	1.0	
**Unmarried**	20	45	1.38	[0.78,2.42]
Accessibility to public services				
**No**	12	51	0.68	[0.35,1.31]
**Yes**	146	423	1.0	

* p < 0.05

We observed no significant between group differences in tobacco exposure prior to or during pregnancy, or in the frequency of urinary tract infections (Table 2). However hypertension (OR, 1.53; 95% CI, 0.93–2.53) and calcium supplementation (OR, 1.86; 95% CI, 0.97–3.56) during pregnancy were marginally significant.

**Table 2:** Tobacco exposure, morbidity during pregnancy and prenatal care characteristics of mothers of low and normal birthweight infants

Variable	Birthweight		OR	95% CI
	**<2500 grs (*n = 158*)**	**≥ 2500 grs (*n = 474*)**		
Smoking before pregnancy				
**No**	126	384	1.0	
**Yes**	32	90	1.09	[0.70,1.71]
Smoking during pregnancy				
**No**	149	451	1.0	
**Yes**	9	23	1.19	[0.53,2.66]
Hypertension during pregnancy				
**No**	131	416	1.0	
**Yes**	27	58	1.53	[0.93,2.53]
Urinary tract infection during pregnancy				
**No**	100	318	1.0	
**Yes**	58	156	1.22	[0.83,1.77]
Calcium supplementation				
**Yes**	146	409	1.0	
**No**	12	65	1.86	[0.97,3.56]
Iron supplementation				
**Yes**	64	216	1.0	
**No**	94	258	1.20	[0.83,1.74]
Prenatal care				
**Yes**	150	454	1.0	
**No**	8	20	1.07	[0.45,2.52]

Reproductive characteristics, including maternal weight, previous preterm-birth, and previous LBW infants, were significantly different between mothers of LBW and normal birthweight infants (Table 3).

**Table 3:** Reproductive characteristicsof mothers of low and normal birthweight infants

Variable	Birthweight		OR	95% CI
	**<2500 grs (*n = 158*)**	**≥ 2500 grs (*n = 474*)**		
Parity				
**Multiparous**	92	284	1.0	
**Primiparous**	66	190	1.04	[0.73,1.51]
Maternal weight (kgs)				
**<48**	54	107	1.61*	[1.09,3.84]
**49–54**	37	120	0.99	[0.58,1.67]
**55–60**	37	119	1.0	
**>60**	30	128	0.75	[0.43,1.29]
Previous^a ^preterm birth				
**No**	58	213	1.0	
**Yes**	34	71	1.75*	[1.06,2.90]
Previous low birth weight^a^				
**No**	66	246	1.0	
**Yes**	26	38	2.55*	[1.44,4.50]
Previous abortion^a^				
**No**	67	222	1.0	
**Yes**	25	62	1.33	[0.77,2.28]

*p <0.05

^a^Only applicable for women with more than one pregnancy

Multivariate analysis showed that low socioeconomic level was the most important risk factor for LBW (Table 4). Using model 1, the OR was 2.57 (95% CI, 1.19–5.58) after adjustments for maternal age, education, marital status, and accessibility to public services. When adjustments for prenatal care, tobacco exposure, and morbidity during pregnancy were also included (model 2), we obtained an OR of 2.45 (95% CI, 1.13–5.36) for low socioeconomic level as a risk factor for LBW (Table 4). When we also included adjustments for reproductive variables (model 3), we obtained an OR of 2.68 (95% CI, 1.19–6.03) for low socioeconomic level as a risk factor for LBW (Table 4).

**Table 4:** Adjusted odds ratios for LBW infants by socioeconomic level among women of Mexico City

**Variable**	**Model 1**		**Model 2**		**Model 3**	
	OR	95% CI	OR	95% CI	OR	95% CI
**Socioeconomic level**						
Medium	1.14	[0.75,1.74]	1.16	[0.76,1.77]	1.12	[0.72,1.74]
Low	2.57	[1.19,5.58]	2.45	[1.13,5.36]	2.68	[1.19,6.03]
**Maternal age (years)**						
<19	0.74	[0.43,1.25]	0.74	[0.43,1.26]	0.57	[0.32,1.03]
>30	1.17	[0.70,1.98]	1.09	[0.64,1.84]	1.34	[0.76,2.38]
**Maternal education (years)**						
< 7	1.21	[0.58,2.56]	1.11	[0.52,2.40]	1.05	[0.47,2.34]
7–9	1.29	[0.63,2.62]	1.23	[0.59,2.55]	1.39	[0.66,2.94]
10–12	1.56	[0.78,3.13]	1.61	[0.79,3.27]	1.82	[0.87,3.77]
**Unmarried**	0.99	[0.49,2.01]	1.08	[0.52,2.28]	0.96	[0.44,2.08]
**No accessibility to public services**	0.68	[0.34,1.33]	0.58	[0.29,1.17]	0.56	[0.27,1.14]
**Smoking before pregnancy**			0.99	[0.57,1.72]	1.07	[0.60,1.90]
**Smoking during pregnancy**			1.09	[0.43,2.78]	1.14	[0.43,2.99]
**Hypertension**			1.61	[0.95,2.73]	1.55	[0.90,2.68]
**Urinary tract infection**			1.23	[0.83,1.83]	1.14	[0.76,1.71]
**No calcium supplementation**			1.98	[1.01,3.87]	2.30	[1.14,4.63]
**No iron supplementation**			1.23	[0.84,1.80]	1.32	[0.89,1.96]
**No prenatal care**			1.15	[0.47,2.87]	1.27	[0.52,3.19]
**Primiparous**					1.73	[1.05,2.83]
**Pre-gestational weight(kgs)**						
<48					2.33	[1.33,4.08]
49–54					1.28	[0.71,2.31]
>60					1.32	[0.74,2.36]
**Previous preterm birth^a^**					2.95	[1.04,8.38]**
**Previous low birth weight^a^**					2.61	[1.36,5.04]

**Previous abortion^a^**					0.52	[0.17,1.63]

^a^only applicable for women with more than one pregnancy.

## Discussion

LBW is a public health problem linked to lack of equity in populations. Despite efforts to decrease the proportion of newborns with LBW, success has been quite limited, and the problem persists in both developing and developed countries [28]. In recent years, studies focused on explaining how social factors influence this problem have shown that populations with greater inequities have a greater proportion of newborns with LBW [25]. These inequities are caused by both social conditions of populations and gender differences. Although these differences are not explicit and conclusive, they are revealed by social indicators such as access to health care services, occupation, income, education, and social exclusion or isolation.

Differences found in the results about the effect of socioeconomic factors on LBW are probably due to the use of different socioeconomic indicators. It should be pointed out, however, that obtaining information that accurately reflects social and economic characteristics can be difficult, leading to the generation of proxy variables. Thus, education has been used as a proxy variable of social class, and occupation has been used as a proxy of socioeconomic status [29]. In addition, studies performed in European countries have used education as a proxy for socioeconomic level [29,30]. A recent Mexican study, however, found no association between LBW and household infrastructure (including lack of indoor sanitation or water facilities and lack of electricity) when used as a socioeconomic indicator, although this study found that LBW was related mainly to access or utilization of prenatal services and disadvantageous maternal lifestyle behaviors [31].

In our study, we used ownership of goods and having a job as indicators of socioeconomic level in a Mexican urban population with social security. Using these indicators, we found that low socioeconomic level is the most important risk factor for LBW, independent of other factors such as reproductive and nutritional characteristics, smoking, morbidity during pregnancy, and accessibility to health services and prenatal care. These results are similar to those of other studies describing a positive relationship between socioeconomic condition and effects on health [32-34]

It is important to mention that to diminish the possible error of misclassifying we used at least two indicators to construct the socioeconomic level variable (ownership of goods and occupation). However we thought that it will be a non differential error. Although we analyzed other proxies of socioeconomic level, we found that each of them was irrelevant. For example, although education has been used as a proxy of social status, we found that this indicator was not important in our population, perhaps because the women in our study were incorporated into the work force, making exposure to intermediate factors, such as occupational stress and load work, an influence on pregnancy outcomes. Family circumstances and biological processes may also be affected by a wider social context, including cultural and historic issues such as educational opportunities, parent's divorce, unemployment, risk of poverty risk, and risks factors for smoking and obesity.

Although many socioeconomic factors related to LBW have been identified, the specific role of each of them is not known, limiting the ability to use preventive actions in exposed populations. Interventions aimed at reducing the number of LBW infants have had limited success on conditions of the newborn, although some showed benefits in pregnant women [35]. To decrease the incidence of LBW, it is important to consider health services interventions to get better quality of care for pregnant women. Investments in the health and development of the most vulnerable populations, such as pregnant women and children, are important in themselves, because they prepare the context and environment for a more productive and healthy life, with the full development and use of mental and physical human potential [36].

## Conclusion

Heterogeneity among different populations makes findings related to interventions in one population not applicable to others. Thus, it is necessary to design studies that account for the geographic, racial, cultural, social and economic context of each country and specific group. In the case of Mexico, we believe that our results provide information representing a closer approach to the effect of socioeconomic status on LBW. Our results provide a starting point in the search for better indicators for evaluating socioeconomic status in Mexican populations with other social conditions, including suburban, rural, and indigenous populations.

Another aspect to consider within the context of each country is the availability of information. In Mexico, there is no way to obtain uniform information about the socioeconomic characteristics of the population. Information about conditions of different populations is important in designing programs aimed at solving existing inequities. It is a challenge to create a socioeconomic index that will reflect the real living conditions of pregnant women in Mexico. This effort will require including other variables, as well as refining proxy variables.

## Competing interests

The author(s) declare that they have no competing interests.

## Authors' contributions

LPTA, PCC, SFH contributed in the conception and design of the study and statistical analysis. JPVB, ERM reviewed for important intellectual content. All authors participated in the interpretation of data and read and approved the final version to be published.

## Pre-publication history

The pre-publication history for this paper can be accessed here:


